# Serological evidence of exposure to *Rickettsia felis* and *Rickettsia typhi* in Australian veterinarians

**DOI:** 10.1186/s13071-017-2075-y

**Published:** 2017-03-13

**Authors:** Yen Thon Teoh, Sze Fui Hii, Mark A. Stevenson, Stephen Graves, Robert Rees, John Stenos, Rebecca J. Traub

**Affiliations:** 1 Faculty of Veterinary and Agricultural Sciences, The University of Melbourne, Parkville, VIC 3010 Australia; 2 The Australian Rickettsial Reference Laboratory, University Hospital, Geelong, VIC 3220 Australia; 3 Bayer Animal Health, Tingalpa, QLD 4173 Australia

**Keywords:** *Rickettsia*, *Rickettsia felis*, *Rickettsia typhi*, flea-borne spotted fever, murine typhus, veterinarian, Australia

## Abstract

**Background:**

*Rickettsia felis* and *Rickettsia typhi* are emerging arthropod-borne zoonoses causing fever and flu-like symptoms. Seroprevalence and risk factors associated with exposure to these organisms was explored in Australian veterinarians.

**Methods:**

One hundred and thirty-one veterinarians from across Australia were recruited to participate in a cross-sectional survey. Veterinarians provided a single blood sample and answered a questionnaire on potential risk factors influencing their exposure to *R. felis* and *R. typhi*. Indirect microimmunofluorescence antibody testing (IFAT) was used to identify evidence of serological exposure of the participants to *R. felis* and *R. typhi*. Results were analyzed and a logistical regression model performed to predict risk factors associated with seropositivity.

**Results:**

In total, 16.0% of participants were seropositive to *R. felis*, 4.6% to *R. typhi* and 35.1% seropositive to both, where cross-reactivity of the IFAT between *R. felis* and *R. typhi* precluded a definitive diagnosis. Veterinarians residing within the south-eastern states of Victoria and Tasmania were at a higher risk of exposure to *R. felis* or generalised *R. felis* or *R. typhi* exposure. Older veterinarians and those that recommended flea treatment to their clients were found to be significantly protected from exposure.

**Conclusions:**

The high exposure to *R. felis* amongst veterinary professionals suggests that flea-borne spotted fever is an important cause of undifferentiated fever conditions that may not be adequately recognized in Australia.

## Background

*Rickettsia felis* is a bacterial pathogen and the etiological agent of flea-borne spotted fever (FBSF) or cat flea typhus, cases of which have been described in many parts of the world including Europe [1], the Americas [2], Asia [3] and Oceania [4]. Human infection results from transmission through an infected arthropod vector, typically fleas infecting a bite site with rickettsiae; the resulting infection is typically characterized by a series of non-specific symptoms including pyrexia, maculopapular rash, eschar, myalgia, arthralgia, headache and fatigue [5].

The biological vector for *R. felis* is the cat flea, *Ctenocephalides felis* [6], although it has also been found in other arthropods. Rickettsiae are generally maintained within reservoir hosts, typically mammals, and their associated arthropod vectors [7]. Efforts to identify a vertebrate biological reservoir for *R. felis* have so far remained unresolved. While *R. felis* DNA has been detected in cat [8], dog [9] and opossum [10] blood, successful culture of the organism from mammalian blood has yet to be achieved. In *C. felis*, *R. felis* is maintained for up to 12 generations in the absence of a blood meal [11].

A number of rickettsial organisms have been described in Australia, including *Rickettsia australis* (causing Queensland tick typhus), *Rickettsia honei* (causing Flinders Island spotted fever), *Rickettsia honei marmionii* (causing Australian spotted fever), *Rickettsia typhi* (causing murine typhus), *Orientia tsutsugamushi* (causing scrub typhus) and *Coxiella burnetti* (causing Q fever) [12]. Each can cause fever and flu-like symptoms, are spread by the bite of an infected arthropod and interact with Australian wildlife in sylvatic cycles. Some species, such as *R. typhi*, display serological cross-reactivity with *R. felis*, presenting a diagnostic challenge requiring concurrent testing against both *R. felis* and *R. typhi* antigen to establish an aetiology [4]. Of the *R. felis*-like species, the URRWXCal2 (Cal2) variant is predominant in *C. felis felis* fleas in Australia [13].

Pet ownership is widespread in Australia, with dog and cat ownership estimated at 36% and 23% respectively [14]. Dogs in particular have been implicated in potentially contributing to the life-cycle of *R. felis*, with molecular detection of the *R. felis omp*B gene in the blood of 9% of pound dogs in south east Queensland [15] and 2.3% of indigenous community dogs in the Northern Territory [13]. Both dogs and cats are well known to harbor ectoparasites, with *C. felis felis* being the dominant flea species [16], from which *R. felis* has also been isolated [17, 18].

The first reported Australian cases of *R. felis* occurred in Victoria [19] in a family living in metropolitan Melbourne, Victoria, that had received two flea-ridden kittens from a farm in Lara, Victoria. Since then, multiple other clinical cases of human infection in patients in New South Wales, Queensland, South Australia, Tasmania and Western Australia have been confirmed [4]. Risk factors for exposure to *R. felis* however were not described. In a seroepidemiological study in Spain, *R. felis* infection was associated with high risk occupations involving working outdoors, contact with animals or potential contact with rodents [20]. In Colombia, gender, home location and age were associated with *R. felis* exposure [21].

In small animal clinical practice, exposure to flea-ridden animals is a potential occupational hazard for veterinarians. Approximately 10,000 veterinarians are employed in Australia [22], a proportion of whom will have contact with animals as part of their job. Due to this potential, veterinarians of Australia are the focus of this study which aims to determine the seroprevalence and risk factors for exposure *R. felis*.

## Methods

### Participant selection

Veterinarians (*n* = 131) were recruited at the Australian Veterinary Association Pan-Pacific conference held in Brisbane (May, 2015) and at the University of Melbourne (December, 2015). Selection was opportunistic, with consenting healthy individuals invited to answer a questionnaire and provide a blood sample on a voluntary basis. Serology testing results were made available to the participants.

### Survey

A questionnaire was designed to collect information from participants on personal demographics and potential risk factors contributing to *R. felis* infection; this included age, gender, location, potential for exposure to different animals in the workplace and at home, knowledge on *R. felis*, attitudes towards flea control in companion animals and any recent disease symptoms. Responses were digitalized, reversibly de-identified, and stored on a password protected computer.

### Blood sample collection

Samples were collected by either a registered medical professional (doctor or nurse) or a certified venepuncturist. Approximately 8 ml of blood was taken from the median cubital vein into serum separator tubes which underwent centrifugation at 4000× *g* for 5 min, and the separated sera stored at -20 °C until processed.

### Culture to obtain antigen

Antigen culture and IFAT was performed at the Australian Rickettsial Reference Laboratory, Geelong, Australia. The L929 cell line was selected to establish culture of the rickettsial organisms tested in this study. Once a confluent monolayer was achieved, live *R. felis* and *R. typhi* cultures were revived from -80 °C and used to infect separate flasks. Leibovitz-15 media (GIBCO, Rockville, MD, USA) supplemented with 10% foetal calf serum, 2 mM L-glutamine and 5% tryptose phosphate broth was used to maintain *R. felis*. RPMI media (GIBCO), supplemented with 10% foetal calf serum and 2 mM L-glutamine was used to maintain *R. typhi*. Infection levels were monitored using a semi-quantitative qPCR, with species confirmation verified using PCR and DNA sequencing (Australian Genomic Research Facility Ltd., Australia); both molecular techniques were based on the citrate synthase (*glt*A) gene [23].

Infected cell monolayers were harvested by physical detachment and heat inactivated at 56 °C for 30 min. Differential centrifugation at 3000× *g* for 10 min at room temperature was used to separate the host cell material from the rickettsiae; this pelleted *Rickettsia* was then resuspended in PBS.

### Immunofluorescence antibody testing

The reference method for the diagnosis of *R. felis* infection is the indirect microimmunofluorescence antibody test (IFAT), a serological test detecting antibodies developed after exposure. Due to shared epitopes, some of which may have been gained from horizontal gene transfer [24], serological cross-reactivity is often noted between *R. felis*, widely considered to be a spotted fever group (SFG) or transitional group *Rickettsia*, with others from the closely related typhus group (TG), such as *R. typhi* [25].

Working concentrations of rickettsial antigen were determined by comparing the fluorescence of serial doubling dilutions of *R. felis* and *R. typhi* on the IFAT. Rickettsial antigen of *R. felis* and *R. typhi* was spotted on 40-well slides (Scientific Device Laboratory, Des Plaines, IL, USA), air-dried and fixed in 100% acetone for 2 min. Serum samples were diluted in 2% casein in PBS at 1:128, and 2-fold serial dilutions we prepared onward as required until the end-point titer was determined. Positive and negative controls were included in each assay run. Slides were incubated at 35 °C for 40 min in a humidified environment, washed in 1/10 PBS, and air-dried. A fluoresceinisothiocyanate (FITC)-labelled goat anti-human immunoglobulin IgG (H + L) (Kirkegaard & Perry Laboratories, Gaithersburg, USA) diluted at 1:1000 was then spotted on each well, and slides incubated for a further 35 °C for 40 min. Following the final washing, the slides were air-dried, covered and stored in a dark environment at 4 °C until read.

Each well was visualized by fluorescence microscopy to the end-point dilution, with a minimum dilution of 1:128 required to deem a sample as reactive. Readings were repeated by a second independent observer to control bias, with a third independent observer recruited to resolve any discrepancies.

Exponentially increasing dilutions were standardized to a linear scale and rickettsial exposure definitively attributed to participants with a preferential serological reactivity at a minimum four-fold dilution difference against one organism compared to the other. Patient samples that tested within this limit were not allocated to a group and were thus classified as indeterminate samples representing mixed infections, reactivity from other related rickettsiae or older infections with lower serological reactivities.

### Data analysis

Results were entered and collected using a spreadsheet application (Libreoffice calc). R statistics software was used to read and analyze the data. Animal exposures were grouped into categories (companion, large and exotic) and location was designated metropolitan or rural based on distance of the participant from the centre of a major city (50 km from Sydney, Melbourne, Brisbane, 40 km from Perth, Adelaide, 30 km from Canberra, Hobart, Darwin). The distribution of the participants was compared with that previously determined by the Australian Bureau of Statistics (ABS) in 2015 [22].

Exploratory analyses were performed using the *epiR* and *epitools* packages, with comparisons made between patients serologically testing preferentially to *R. felis* or *R. typhi*, as well as a category for general rickettsial reactivity including either preferentially reactive sera as well as indeterminate sera. Univariate analyses using probability ratio analyses were compiled from information on risk factors as determined by the questionnaire, and biological risk factors meeting the odds-ratio criteria with a *P*-value less than 0.2 were selected for inclusion in the multivariate model. Models were developed using the glm function by backwards elimination based on potential risk factors identified in the exploratory data analysis. Graphics were generated using the *ggplot2* package, with map data from the GADM database.

## Results

**Fig. 1:**
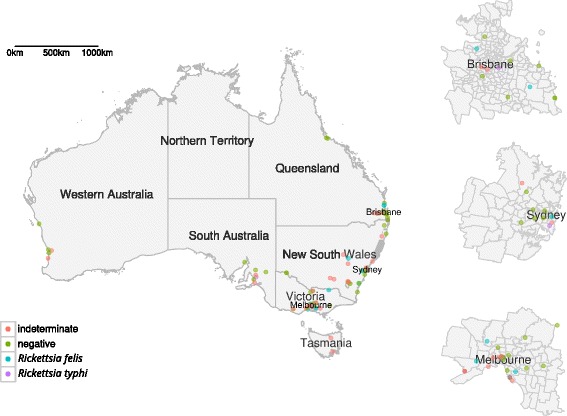
Map of exposures and their associated serological testing exposures

**Fig. 2:**
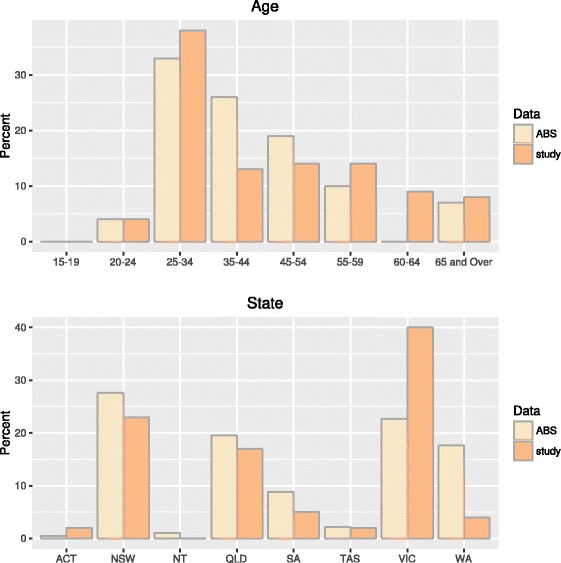
Comparisons of study participants to veterinary population as gathered by the Australian Bureau of Statistics (ABS)

A total of 131 veterinarians were recruited for this study (Fig. 1), with a distribution based on age and state approximately representative of the distribution of veterinarians across Australia (Fig. 2) with the exception of the Northern Territory, from which no veterinarians were recruited. Younger participants (20–39 years) and middle-aged participants (40–59 years), on average, spent more hours in private practice (22 and 24 h per week, respectively) compared with 17.5 h per week for older participants.

Awareness of *R. felis* and its associated zoonotic risk was assessed, with only 72 of 127 (55.8%) veterinarians who recorded a response to this question stated they were aware of the organism. While the majority of the 127 respondents to the question (122 or 96.1%) were of the opinion that fleas posed a risk to animals, only 65 (51.2%) thought that fleas also posed a risk to humans. Of the 73 *Rickettsia*-exposed positive participants, 27 of 71 respondents (38.0%) recorded no recent (within the last 3 months) flea bites.

IFAT attributed twenty-one (16.0%) participants to *R. felis* exposure and six (4.6%) to *R. typhi* exposure from a specifically preferential reaction to the respective antigen. Forty-six (35.1%) veterinarians had a serological reaction to either antigen at a level which was within two serial dilutions to one another, and so a diagnosis was not able to be definitively made based solely on serology. Fifty-eight (44.3%) tested negative by IFAT, suggesting no recent exposure to either organism.

### Risk factors

Univariate analyses indicated that sex, metropolitan/rural location and outdoor activity were not significant for exposure to *R. felis P* > 0.2. Exposure to companion animals was not a significant predictor of *R. felis* exposure when tested in isolation (*P* = 1; OR: 1.27, 95% CI: 0.25–6.55). A confounding influence from flea-treatment was noted with Mantel-Haenszel testing: [*t* = 0.006, *P* = 0.938 (OR (crude): 1.26, 95% CI: 0.24–6.49; OR (M-H): 1.91, 95% CI: 0.35–10.31; crude: M-H: 0.66)]. Once adjusted, a non-significant but stronger trend was observed amongst veterinarians that had contact with companion animals whilst concurrently not treating their own animals for fleas (*P* = 0.5; OR: 1.81, 95% CI: 0.43–7.64). Further non-significant positive trends were observed for reported disease symptoms (including rashes, headaches and fevers) potentially associated with rickettsial exposure.

Multivariate logistical regression converged for data on *R. felis* or *R. typhi* (including indeterminate) infection and *R. felis* infection is displayed in Tables 1 and 2; analysis on the *R. typhi* subset was not successful in producing a model due to low numbers of positives within our study population (Table 3). Participants were divided into categories according to age (20–39, 40–59 (reference category), 60+ years) and region [southeast including Victoria and Tasmania, northeast including Queensland, east including New South Wales and Canberra and south/west including Western Australia and South Australia (reference category)]. Older veterinarians above the age of 60 were at a significantly decreased risk of exposure to *R. felis* (*t* = -2.095, *P* = 0.04; OR: 0.756, 95% CI: 0.582–0.982) or generalized *R. felis* or *R. typhi* exposure (*t* = -2.147, *P* = 0.034; OR: 0.752, 95% CI: 0.579–0.975). Veterinarians recommending flea treatments to clients were also at a significantly decreased risk of exposure to generalized *R. felis* or *R. typhi* exposure (*t* = -2.034, *P* = 0.044; OR: 0.611, 95% CI: 0.38–0.982). Conversely, participants working in the southeastern Australian states of Victoria or Tasmania were at an increased risk of *R. felis* exposure (*t* = 1.808, *P* = 0.075; OR: 1.381, 95% CI: 0.973–1.96).

**Table 1:** Multivariate risk factor analysis of exposure to *Rickettsia felis* or *R. typhi*

	Population	Exposed	Coefficient (SE)	*t*	*P*	OR (95% CI)
Constant			0.993 (0.291)	3.41	0.001	
Age						
20–39 years	64	42	0.106 (0.101)	1.051	0.295	1.112 (0.912–1.356)^a^
40–59 years	42	24	Reference			
60+ years	23	6	-0.285 (0.133)	-2.147	0.034	0.752 (0.579–0.975)
State						
SA or WA	12	5	Reference			
NSW or ACT	33	16	-0.040 (0.163)	-0.244	0.808	0.961 (0.698–1.323)
QLD	22	10	-0.046 (0.175)	-0.263	0.793	0.955 (0.678–1.346)
VIC or TAS	55	36	0.093 (0.157)	0.591	0.555	1.097 (0.807–1.493)
Recommends flea treatment to clients						
No	5	5	Reference			
Yes	123	66	-0.493 (0.242)	-2.034	0.044	0.611 (0.38–0.982)

*Abbreviations*: *CI* confidence interval, *OR* odds ratio, *SE* standard error

^a^Interpretation: compared with participants from the reference category (those between the ages of 40–59), after adjusting for the effect of location (state) and whether they recommended flea treatment to clients, participants aged 60+ had a 1.330 (0.752^-1^; CI: 1.026–1.727) times lower odds of exposure

**Table 2:** Multivariate risk factor analysis of exposure to *Rickettsia felis*

	Population	Exposed	Coefficient (SE)	*t*	*P*	OR (95% CI)
Constant			0.111 (0.177)	0.625	0.534	
Age						
20–39 years	34	12	0.033 (0.112)	0.294	0.769	1.033 (0.83–1.286)
40–59 years	27	9	Reference			
60+ years	17	0	-0.28 (0.134)	-2.095	0.04	0.756 (0.582–0.982)
State						
SA or WA	7	0	Reference			
NSW or ACT	21	4	0.132 (0.185)	0.716	0.477	1.142 (0.794–1.641)
QLD	15	3	0.151 (0.194)	0.78	0.438	1.163 (0.796–1.699)
VIC or TAS	32	13	0.323 (0.179)	1.808	0.075	1.381 (0.973–1.96)

*Abbreviations*: *CI* confidence interval, *OR* odds ratio, *SE* standard error

**Table 3:** Multivariate risk factor analysis of exposure to *Rickettsia typhi*

	Population	Exposed	Coefficient (SE)	*t*	*P*	OR (95% CI)
Constant			0.182 (0.062)	2.923	0.005	
Age						
20–39 years	24	2	-0.098 (0.086)	-1.144	0.257	0.906 (0.765–1.073)
40–59 years	22	4	Reference			
60+ years	17	0	-0.182 (0.094)	-1.93	0.058	0.834 (0.693–1.003)

*Abbreviations*: *CI* confidence interval, *OR* odds ratio, *SE* standard error

## Discussion

This study is the first to demonstrate natural exposure to *R. felis* in 16.0%, *R. typhi* in 4.6% and potential exposure to either or both in a further 35.1% of Australian veterinarians. This builds upon the evidence that human exposure, as demonstrated in earlier studies in Spain [20] and Colombia [21], where *R. felis* is ubiquitous in areas where *R. felis* Cal2 has been detected in fleas, notably *C. felis felis*. Infection with *R. felis* in Australia has been previously reported in 20% of companion animal fleas in Brisbane, Sydney and Melbourne [17] and up to 36% in regional centers in Western Australia [18]. Veterinarians in clinical practice are regularly exposed to animals, particularly companion animals (cats and dogs), which may act as potential hosts for ectoparasites. Trends were also seen when observing *R. felis* and *R. typhi* reactive participants in combination, which is not surprising as both organisms share vectors and hosts [2].

In this study, *R. felis* exposure was noted across each of the Australian states tested with proportionally the highest participant count from Victoria. We were able to demonstrate an increased likelihood of exposure in veterinarians working within the states of Victoria and Tasmania (β = 0.323; SE = 0.179; *t* = 1.808; *P* = 0.075). Both are coastal south-eastern states and have a temperate climate. While state-based flea infection rates of *R. felis* have not been thoroughly investigated within Australia, it may be a reflection of higher *R. felis*-flea infection rates in these cooler climates, as reported previously in other parts of the world [26]. It should be noted that this study was centered on metropolitan areas, whereas previous studies on *R. felis* infection rates in Australia compared rural and metropolitan flea populations [9, 18].

While epizoonotic associations are typically observed in areas with a warm temperature with precipitation, high temperatures have been noted to affect the survival of fleas as well as vector-borne disease transmission [27]. Given the unusual growth characteristics of *R. felis* requiring an optimal growth temperature lower than that typical of other rickettsiae at 28 °C [28], a link between Victoria and Tasmania with their moderate and cool temperate climates and *R. felis* seropositivity is plausible.

Characterization of the epidemiology of FBSF has been complicated by the wide-spread nature of incidental exposure, compounded by a typically long-lived antibody response and non-specific symptoms characteristic of other fever-causing conditions [5]. It is clear from this study that some of these veterinarians had been exposed to *R. felis* in the past, but no veterinarians had reported clinical symptoms matching the disease syndrome [5] and none had been medically diagnosed. This is supportive of exposure being common but with a mild self-resolving flu-like manifestation rather than severe clinical FBSF [19, 20].

Older participants (aged 60+) had a 1.323 times lower odds of exposure to *R. felis* (*t* = -2.095, *P* = 0.040; OR: 0.756; CI: 0.582–0.982), 1.202 times lower to *R. typhi* (*t* = -1.93, *P* = 0.058; OR: 0.834, CI: 0.693–1.003), and 1.330 times lower to either *R. felis* or *R. typhi* (t = -2.147; *P* = 0.034; OR: 0.752; CI: 0.579–0.975) exposures, which is consistent with the findings of Hidalgo et al. in a similar study in Spain [21]. In our study, actively working older participants spent less time (17.5 h) in private practice compared with their younger and middle-aged counterparts (22 and 24 h, respectively). This lower clinical exposure is likely reflected in the changing likelihood of rickettsial exposure.

All five veterinarians who indicated that they did not recommend flea-treatment to clients were positive to rickettsial exposure (two to *R. felis*, three to indeterminate exposure). This result is suggestive of a 1.637 times reduction in odds of exposure to FBSF or MT for veterinarians that recommended their clients treat their pets for fleas ($\beta$ = -0.493; SE = 0.242; *t* = -2.034; *P* = 0.044). Attitudes towards regular flea prophylaxis may effectively function as a reliable predictor for exposure to *R. felis* with intrinsic ties to the potential for exposure of the general population to flea-borne zoonoses from flea-ridden pets and animals. A potential lack of awareness of recent flea exposure and being bitten by a flea were also trends seen in the data, in which 29 *Rickettsia* exposed participants reported with certainty that they had not been recently bitten, highlighting that people may not realize that they have been exposed to flea bites or inhaled flea feces [29] and thus the zoonotic vector-borne organisms fleas harbor.

In our study, no statistically significant risk factors were able to be linked between exposure to either *R. felis* or *R. typhi* and contact with companion animals or with fleas. This may be a reflection of the widespread, ubiquitous exposure amongst the veterinary populace tested to these factors (e.g. companion animals and the fleas associated with them). There is an ongoing issue with *R. felis* serology is cross-reactivity with TG rickettsiae (e.g. *R. typhi*) antibodies [30] impeding acquisition of a definitive serodiagnosis.

A validated protocol used for diagnostic testing [4] was followed where serology was performed concurrently on each sample tested against both *R. felis* and *R. typhi*, with a positive result considered only when a sample tested greater or equal to two serial dilutions (a four-fold increase) of one antigen over the other. This ensured rigor of the classification of the *R. felis*- and *R. typhi*-exposed patients as high compared to previous serosurveys which utilized protocols that used lower cut-off titers [31] whilst being able to confidently classify the etiological agent [4]. Consequently, the number of participants testing positive for an indeterminate rickettsial infection was produced. It is likely that a proportion of these participants were only exposed to either *R. felis* or *R. typhi* infections or alternatively as mixed infections, where a patient may have been exposed to both *R. felis* and *R. typhi*. An improvement in the specificity of the test used (e.g. by using cross-adsorption or Western blotting) may result in clearer data on individual exposure status.

## Conclusions

In Australia, veterinarians are considered at the forefront of diagnosis, treatment and prevention of zoonotic diseases from companion animals to their owners and the general public. Given the low awareness of *R. felis* and FBSF amongst our participants, improved education of veterinarians and in turn pet owners is needed. In turn, communication between medical, veterinary and diagnostic laboratory professions is also imperative for the diagnosis and prevention of this common zoonosis. The reported clinical cases of FBSF within the Australian population [4] coupled with high exposure amongst veterinary professionals suggests that FBSF is an important cause of undifferentiated fever conditions that may not be adequately recognized and potentially treated.

## Abbreviations

ARRL: Australian Rickettsial Reference Laboratory
Cal2: *Rickettsia felis* URRWXCal2
CI: confidence interval
FBSF: flea-borne spotted fever
FITC: fluoroscein isothiocyanate
GADM: global administrative regions
IFAT: immunofluorescence antibody testing
IgG: immunoglobulin G
Inf: infinity
M-H: Mantel-Haenszel correction
MT: murine typhus
PBS: phosphate buffered saline
SE: standard error
SFG: spotted fever group
TG: Typhus group
β: beta coefficient
